# Gemcitabine enhances pharmacokinetic exposure of the major components of Danggui Buxue Decoction in rat via the promotion of intestinal permeability and down-regulation of CYP3A for combination treatment of non-small cell lung cancer

**DOI:** 10.1080/13880209.2023.2246500

**Published:** 2023-08-22

**Authors:** Xin Xu, Xi-yang Sun, Ming Chang, Zhao-liang Hu, Ting-ting Cheng, Tai-jun Hang, Min Song

**Affiliations:** a Key Laboratory of Drug Quality Control and Pharmacovigilance, Ministry of Education, China Pharmaceutical University, Nanjing, China; b Department of Pharmaceutical Analysis, China Pharmaceutical University, Nanjing, China

**Keywords:** Traditional Chinese medicine, bioavailability, metabolism, herb–drug interaction

## Abstract

**Context:**

Danggui Buxue Decoction (DBD), a traditional Chinese medicine formula, has the potential to enhance the antitumor effect of gemcitabine in non-small cell lung cancer (NSCLC) treatment by increasing gemcitabine’s active metabolites. However, whether gemcitabine affects the pharmacokinetics of DBD’s major components remains unclear.

**Objective:**

This study evaluates the herb–drug interaction between DBD’s major components and gemcitabine and validates the underlying pharmacokinetic mechanism.

**Materials and methods:**

The pharmacokinetics of 3.6 g/kg DBD with and without a single-dose administration of 50 mg/kg gemcitabine was investigated in Sprague-Dawley rats. The effects of gemcitabine on intestinal permeability, hepatic microsomal enzymes in rat tissues, and CYP3A overexpressing HepG2 cells were determined using western blot analysis.

**Results:**

The combination of gemcitabine significantly altered the pharmacokinetic profiles of DBD’s major components in rats. The *C*_max_ and AUC of calycosin-7-*O*-β-d-glucoside notably increased through sodium-glucose transporter 1 (SGLT-1) expression promotion. The AUC of ligustilide and ferulic acid was also significantly elevated with the elimination half-life (*t*_1/2_) prolonged by 2.4-fold and 7.8-fold, respectively, by down-regulating hepatic CYP3A, tight junction proteins zonula occludens-1 (ZO-1) and occludin expression.

**Discussion and conclusions:**

Gemcitabine could modulate the pharmacokinetics of DBD’s major components by increasing intestinal permeability, enhancing transporter expression, and down-regulating CYP3A. These findings provide critical information for clinical research on DBD as an adjuvant for NSCLC with gemcitabine and help make potential dosage adjustments more scientifically and rationally.

## Introduction

Danggui Buxue Decoction (DBD) is a traditional Chinese medicine (TCM) formula that was first recorded in Nei Wai Shang Bian Huo Lun written by Li Dongyuan (Jin Dynasty, more than 800 years ago) as a blood supplement. DBD is composed of Radix *Astragalus mongholicus* Bunge (Fabaceae) (RA) and Radix *Angelica sinensis* (Oliv.) Diels (Apiaceae) (RAS) at the ratio of 5:1, which has been used for the clinical treatment of anaemia for hundreds of years (Wang and Liang 2010; Shi et al. 2020).

Lung cancer is the leading cause of malignant tumour deaths in China (Hong et al. 2015; Gao et al. 2020), with non-small cell lung cancer (NSCLC) being the most lethal type, characterized by a meager 5-year survival rate (Torre et al. 2015; Wood et al. 2015). Chemotherapeutic agents, such as gemcitabine and paclitaxel (Binenbaum et al. 2015), are commonly used treatments for NSCLC. However, their side effects cannot be avoided, including bone marrow suppression, gastrointestinal discomfort and hepatorenal response (Bayman et al. 2014; Schild and Vokes 2016). Clinical practices have shown that DBD enhances immune function in NSCLC patients, exhibiting antioxidant and immunoregulatory effects (Du et al. 2009). Moreover, pharmacological studies have demonstrated that DBD inhibits immune-mediated erythrocytopenia, promotes myelopoietic progenitor cells (Yang et al. 2014), induces T-lymphocytes proliferation and interleukin secretion (IL-2, IL-6 and IL-10), activates extracellular signal-regulated kinase phosphorylation, and increases macrophagocytosis (Gao et al. 2007). Furthermore, previous studies have found that DBD increases the concentration of active metabolite triphosphate gemcitabine in peripheral blood mononuclear cells, enhances the antitumour effect of gemcitabine in rats, and alleviates gemcitabine-induced myelosuppression (Sun et al. 2019; Liu et al. 2021).

Moreover, since TCMs possess distinct mechanisms of action, TCMs could potentially sensitize tumours that are normally resistant to conventional chemotherapy while diminishing their side effects. However, when combined with chemical drugs, TCMs may affect the transporters and the activity of metabolic enzymes, leading to changes in pharmacokinetics and interactions with TCMs *in vivo*. For example, rotundic acid, a classical *Ilex rotunda* Thunb. (Aquifoliaceae)-derived ingredient, exhibited changed pharmacokinetics when verapamil was administered through P-glycoprotein and CYP3A regulation (Shang et al. 2021). Similarly, broad-spectrum antibiotics pretreatment significantly altered the pharmacokinetic behaviours of seven major constituents of Shaoyao-Gancao decoction, showing decreased absorption and inhibited intestinal biotransformation metabolism (Liu et al. 2019). Furthermore, an *in vitro* study has demonstrated that the components of *Angelica sinensis* (Oliv.) Diels (Apiaceae), such as ferulic acid, can enhance the membrane permeability of *Astragalus mongholicus* Bunge (Fabaceae)-derived components formononetin and calycosin in Caco-2 cells (Zheng et al. 2012). For all we know, there is no study on the *in vivo* interaction of the active ingredients of DBD with chemical medicines. Calycosin-7-*O*-β-d-glucoside, ononin, ligustilide and ferulic acid are the four most abundant active ingredients in DBD. Calycosin-7-*O*-β-d-glucoside, ononin and ferulic acid have been reported to have antioxidant (Fu et al. 2014; Zduńska et al. 2018) and immunoregulatory effects (Kilani-Jaziri et al. 2017). Radix *Angelica sinensis* (Oliv.) Diels (Apiaceae) volatile oil, represented by ligustilide, has a definite anticancer effect (Chiu et al. 2017; Lang et al. 2018). Although DBD significantly altered the pharmacokinetic behaviour of the active metabolite dFdCTP of gemcitabine in rats and enhanced its bioavailability *in vivo* (Sun et al. 2019), it is unclear whether gemcitabine can alter the pharmacokinetic behaviours of the active components of DBD.

This study aimed to evaluate the pharmacokinetic behaviours of the major components of DBD in rats combined with gemcitabine. The observed enhancement in exposure prompted us to investigate the underlying interaction mechanism, including intestinal permeability, glucose transport and hepatic enzyme expression. The findings presented in this study will serve as a scientific foundation for the clinical use of DBD as an adjuvant treatment for NSCLC in combination with gemcitabine.

## Materials and methods

### Materials and reagents

**Figure 1.**
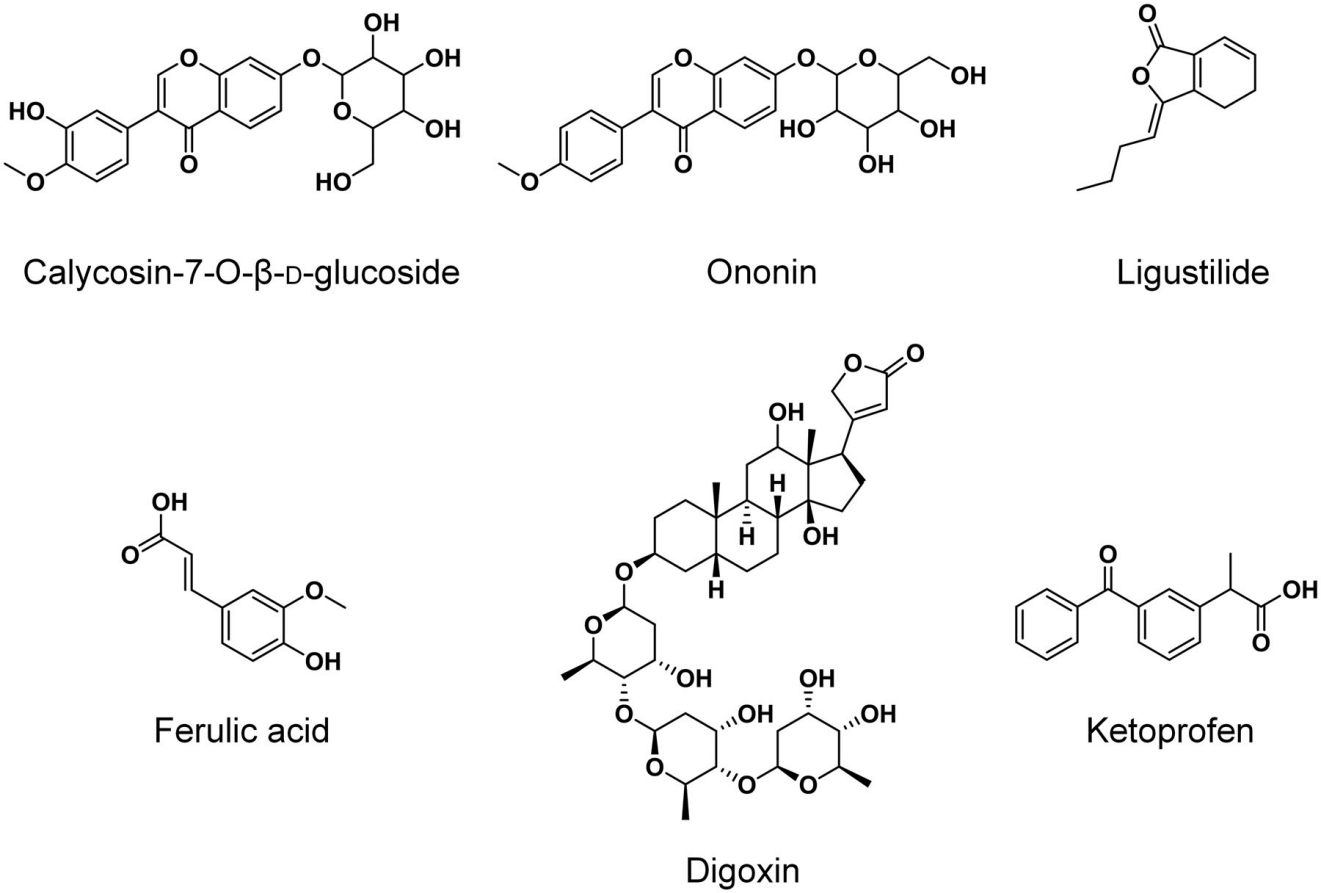
Chemical structures of calycosin-7-*O*-β-d-glucoside, ononin, ligustilide, ferulic acid, digoxin (IS1) and ketoprofen (IS2).

Radix *Angelica sinensis* (Oliv.) Diels (Apiaceae) (RAS, lot no.: 20181101) and Radix *Astragalus mongholicus* Bunge (Fabaceae) (RA, lot no.: 1810004) were purchased from a local TCM store in Nanjing and authenticated by Dr. Xie Guoyong, associate professor in the Department of Chinese Medicine Resources at China Pharmaceutical University, Nanjing, China. Gemcitabine hydrochloride (JX-101170601, purity: 100.2%) was obtained from JARI Pharmaceutical Co., Ltd. (Lianyungang, China). Reference standards of calycosin-7-*O*-β-d-glucoside (DST161107-013, purity: 99.4%), ononin (DST170314-044, purity: 99.3%) and ligustilide (DST170315-007, purity: 98.9%) were purchased from Desite Bio-Technology Co., Ltd. (Chengdu, China). Ferulic acid (J1619083, purity: 99.0%) was purchased from Aladdin Biochemical Technology Co., Ltd. (Shanghai, China). Digoxin (100015-200709, purity: 99.8%) and ketoprofen (100337-200502, purity: 99.9%) were obtained from the National Institute for the Control of Pharmaceutical and Biological Products (Beijing, China). Their chemical structures are presented in Figure 1.

HPLC-grade methanol and acetonitrile were obtained from Tedia (Fairfield, OH). Formic acid and ethanol of analytical grade were purchased from Nanjing Chemical Reagent Co., Ltd. (Nanjing, China). The antibody of sodium-glucose transporter 1 (SGLT-1) was purchased from Cell Signaling Technology (Danvers, MA). Occludin, zonula occludens-1 (ZO-1) and β-actin antibodies were obtained from Santa Cruz (Dallas, TX), and the CYP3A antibody and HRP-conjugated secondary antibodies were purchased from Proteintech (Rosemont, IL). Water was purified using an Explorer purification system (Shanghai, China).

### Apparatus

Thermo Fischer TSQ Quantum Ultra AM mass spectrometry system (equipped with ESI source) and Dionex Ultimate 3000 UPLC system (Thermo Fischer Scientific Inc., Waltham, MA); Sartorius BS21S and BS110S electronic balance (Sartorius Scientific Instruments Corporation, Beijing, China); TGL-16 refrigerated centrifuge (Xiangyi Instrument Corporation, Changsha, China); RE-207B rotary evaporator (Keer Equipment PTY., Ltd., Nanjing, China).

### Preparation of gemcitabine injection and DBD extract

Gemcitabine hydrochloride was weighed accurately and dissolved in normal saline to prepare an isotonic solution with a concentration of 25 mg/mL; then, the injection was filtered for sterilization.

RAS powder (10 g) was extracted by Soxhlet extraction with 10–15 times the volume of ethanol, and the RAS ethanol extract was stored at 4 °C. RA (50 g) and the RAS powder after ethanol extraction were decocted twice in water (1:8, w/v) for 2 h each time. The water extract was evaporated by rotary evaporation under vacuum at 75 °C to a proper volume, and then a triple volume of ethanol was gently mixed and kept in the dark overnight. The mixture was then centrifuged at 1000 rpm for 10 min, and the supernatant was evaporated by rotary evaporation under a vacuum at 45 °C. The residual was freeze-dried, dissolved in pure water, and merged with the RAS ethanol extraction. The final extract was concentrated to a 1.0 g/mL concentration without ethanol (containing extractants of about 0.3 g/mL). The DBD contained calycosin-7-*O*-β-d-glucoside (118.8 ± 14.2 μg/mL), ononin (73.6 ± 2.7 μg/mL), ligustilide (345.8 ± 14.9 μg/mL) and ferulic acid (60.8 ± 3.4 μg/mL) (*n* = 3).

### UPLC–MS/MS-based bioanalytical method conditions

The separation of calycosin-7-*O*-β-d-glucoside, ononin and ligustilide was performed on a Curo-Sil PFP column (4.6 mm × 150 mm, 5 μm) (Phenomenex, Torrance, CA), using a mix solution of methanol–water (5:95, v/v) containing 0.1% formic acid as mobile phase A and methanol as mobile phase B in linear gradient elution mode (A:B): 0 min (70:30) → 6.5 min (10:90) → 8.5 min (10:90) → 8.6 min (70:30) → 10 min (70:30). The flow rate was set at 1.0 mL/min and the column temperature was maintained at 35 °C.

The analysis of ferulic acid was carried out on a BDS HYPERSIL C_18_ column (4.6 mm × 100 mm, 2.4 μm) (Thermo Scientific, Waltham, MA), using water containing 0.01% formic acid as mobile phase A and acetonitrile as mobile phase B in linear gradient elution mode (A:B): 0 min (80:20) → 4.5 min (10:90) → 5.5 min (10:90) → 5.6 min (80:20) → 7.5 min (80:20). The flow rate was 0.65 mL/min and the column temperature was 35 °C.

The TSQ Quantum parameters were optimized and set, as shown in Table 1.

**Table 1.** TSQ Quantum Ultra MRM scan parameters.

Analytes	Polarity	Spray voltage (kV)	Heated capillary temperature (°C)	Sheath gas (psi)	Auxiliary gas (psi)	Collision gas (mTorr)	Precursor ion (*m/z*)	Product ion (*m/z*)	Collision energy (eV)
Calycosin-7-O-β-d-glucoside	+	4	350	40	5	1.2	447.1	285.1	16
Ononin	+	4	350	40	5	1.2	431.1	269.0	15
Ligustilide	+	4	350	40	5	1.2	191.1	91.2	28
Digoxin (IS1)	+	4	350	40	5	1.2	803.4	387.1	37
Ferulic acid	–	3	350	40	5	1.2	193.0	134.0	19
Ketoprofen (IS2)	–	3	350	40	5	1.2	253.0	209.0	10

### Preparation of calibration standards and quality control samples

The stock solutions of calycosin-7-*O*-β-d-glucoside, ononin, ligustilide and ferulic acid were prepared separately in methanol at approximately 500 μg/mL and stored at −20 °C. The stock solutions were serially diluted with methanol to prepare working standard solutions. Calibration standards were prepared by spiking the standard working solutions with blank plasma to obtain final serial concentrations of 0.50–49.7 ng/mL for calycosin-7-*O*-β-d-glucoside, 0.50–49.5 ng/mL for ononin, 5.08–508 ng/mL for ligustilide, and 5.03–503 ng/mL for ferulic acid. Quality control (QC) samples were prepared at concentrations of 1.00, 5.00 and 40.0 ng/mL for calycosin-7-*O*-β-d-glucoside, 1.00, 4.98 and 39.8 ng/mL for ononin, 10.3, 51.7 and 414 ng/mL for ligustilide, and 12.8, 40.9 and 307 ng/mL for ferulic acid from separate stocking solutions.

The internal standard (IS) stock solutions of digoxin and ketoprofen were prepared in methanol at an approximate concentration of 500 μg/mL and diluted to 2.01 μg/mL for digoxin and 501 ng/mL for ketoprofen.

### Sample processing

To each plasma sample in a 2 mL Eppendorf tube, IS solution (50 μL) and methanol (50 μL for calibration or QC working solutions) were added. The mixture was briefly vortexed for 30 s, followed by adding 0.1 mL methanol. The mixture was then vortexed for 3 min and centrifuged at 12,000 rpm for 10 min at 4 °C. An aliquot of 20 μL of the supernatant was injected into the UPLC–MS/MS system for analysis.

### Pharmacokinetic study

Twenty Sprague-Dawley rats (200 ± 20 g, equal number of male and female) were obtained from Shanghai Sippr/BK Lab Animal Co., Ltd. (Shanghai, China) and housed at China Pharmaceutical University Pharmaceutical Animal Experiment Center (Nanjing, China) (temperature: 20–24 °C, humidity: 40–70%). The rats were acclimated to the facilities for five days and fasted for 12 h with free access to drinking water before the experiments commenced. The rats were randomized into two groups, namely DBD and DBD + gemcitabine (DBD + Gem). Rats in both groups were orally administered DBD at a dosage of 3.6 g/kg body weight. Meanwhile, the rats in DBD + Gem group were intravenously injected with gemcitabine via the caudal vein at a dosage of 50 mg/kg body weight, while those in the DBD group were given saline at a dosage of 2 mL/kg body weight. Blood samples were collected into the heparinized tubes at specific intervals (0, 2, 5, 10, 15, 30, 60 and 240 min) and then centrifuged at 3000 rpm for 10 min. After that, the resulting supernatant was transferred and stored at −80 °C in the dark for subsequent analysis.

Animal studies (ethics approval number: 201909010) were approved by the Animal Ethics Committee of China Pharmaceutical University, Nanjing, China. Moreover, experimental procedures complied with the Guide for Care and Use of Laboratory Animals (NIH publication, revised 1996).

### Valuation of intestinal permeability, transporter and hepatic microsomal enzyme

#### Valuation of intestinal permeability and endothelial cell apoptosis

Twelve Sprague-Dawley male rats were randomized into the DBD and DBD + Gem groups to evaluate the histopathological indicators of intestinal permeability. All rats orally received DBD at a dosage of 3.6 g/kg body weight. The rats in the DBD + Gem group were additionally treated with a 50 mg/kg dose of gemcitabine by intraperitoneal injection, whereas the DBD group received saline. After 48 h, the rats were euthanized, and their small intestines were rapidly excised, rinsed with saline, and dried using filter paper. Then, the organs were fixed with formalin solution, embedded in paraffin, sliced and stained with haematoxylin and eosin for an accurate histopathological assessment of intestinal permeability.

TUNEL staining was performed on the paraffin sections of the jejunum and ileum of rats to evaluate the level of apoptosis of the intestinal endothelial cells. The TUNEL cell apoptosis assay kit (Beyotime, Shanghai, China) was employed, following the manufacturer’s instructions. The procedures entailed deparaffinization with xylene, rehydration with graded ethanol and water, and permeabilization with a proteinase K solution. After rinsing with PBS, the sections were stained with TUNEL detection reagent and observed under fluorescence microscopy upon sealing with an anti-fluorescence quencher mounting medium containing DAPI.

#### Investigation of expression of SGLT-1, occludin and ZO-1 in the small intestine and CYP3A expression in the liver by western blot analysis

Protein samples from the small intestine tissues and liver were obtained from the supernatant of homogenized and centrifuged tissue lysates and separated on an 8% SDS polyacrylamide gel electrophoresis before being transferred onto PVDF membranes. The membranes were then blocked with 5% defatted milk in TBST for 1 h and incubated with primary antibodies of SGLT-1 (1:1000), occludin (1:400), ZO-1 (1:400), CYP3A (1:2000) and β-actin (1:400) at a temperature of 4 °C overnight. Afterward, the membranes were rinsed with TBST three-times before being incubated with a diluted secondary antibody (1:2000) for 1 h at room temperature. The density of the bands was visualized and determined by chemiluminescence.

#### Establishment of CYP3A overexpressing HepG2 cell line and investigation of the effect of gemcitabine on CYP3A expression

HepG2 cell line was obtained from Cell Bank, Shanghai Institutes for Biological Sciences of Chinese Academy of Sciences (Shanghai, China). Cells were maintained in DMEM supplemented with 10% FBS (Gibco, Carlsbad, CA) and 1% penicillin/streptomycin (Beyotime, Shanghai, China) at 37 °C in a humidified 5% CO_2_ incubator. To overexpress CYP3A in HepG2 cells, the CYP3A coding sequence was introduced into the CMV-MCS-EF1α-ZsGreen1-PGK-Puro (PHY-028) plasmid vector to construct the overexpression plasmid PHY-028-CYP3A. Following the manufacturer’s instructions, the HepG2 cells were transfected with the plasmid DNA using the ExFect transfection reagent (Vazyme, Nanjing, China). Specifically, the ExFect transfection reagent and plasmid DNA were diluted separately in opti-MEM (Gibco, Carlsbad, CA) without FBS, mixed at a ratio of 2:1, and then added to HepG2 cells for 36 h of culture. Following transfection, HepG2 cells were exposed to gemcitabine at 100 or 200 nM for 12 h. After cell lysis, the changes in CYP3A expression levels in HepG2 cells were determined using western blot analysis.

### Statistical analysis

Pharmacokinetic parameters were calculated by WinNonlin 6.2 (Pharsight, St. Louis, MO). Statistical differences/significance were determined using a two-tailed Student’s *t*-test (two groups) or one-way ANOVA with *post hoc* Bonferroni/Dunnett’s test (three or more groups). *p* < 0.05 means the difference is statistically significant.

## Results

### Method validation

The UPLC–MS/MS methods have been validated following the guideline on bioanalytical method validation outlined in the European Medicines Agency (EMA) publication (EMA 2011).

#### Selectivity, linearity and LLOQs

**Figure 2.**
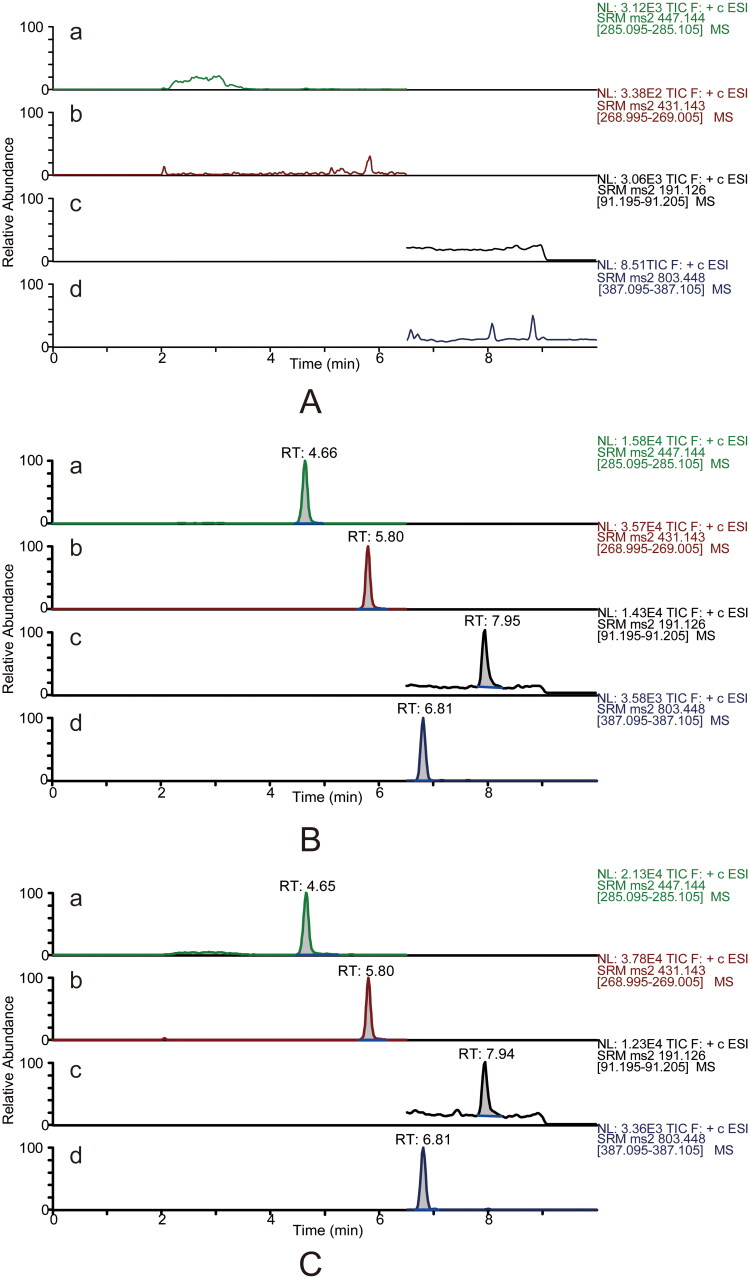
Typical UPLC–MS/MS chromatograms in the positive mode for the quantification of calycosin-7-*O*-β-d-glucoside (a), ononin (b), ligustilide (c) and the internal standard digoxin (d, IS1). Blank plasma sample (A). Blank plasma sample spiked with reference standards (medium level QC sample) and the internal standard (B). The rat plasma sample was collected at 5 min after DBD administration (C).

**Figure 3.**
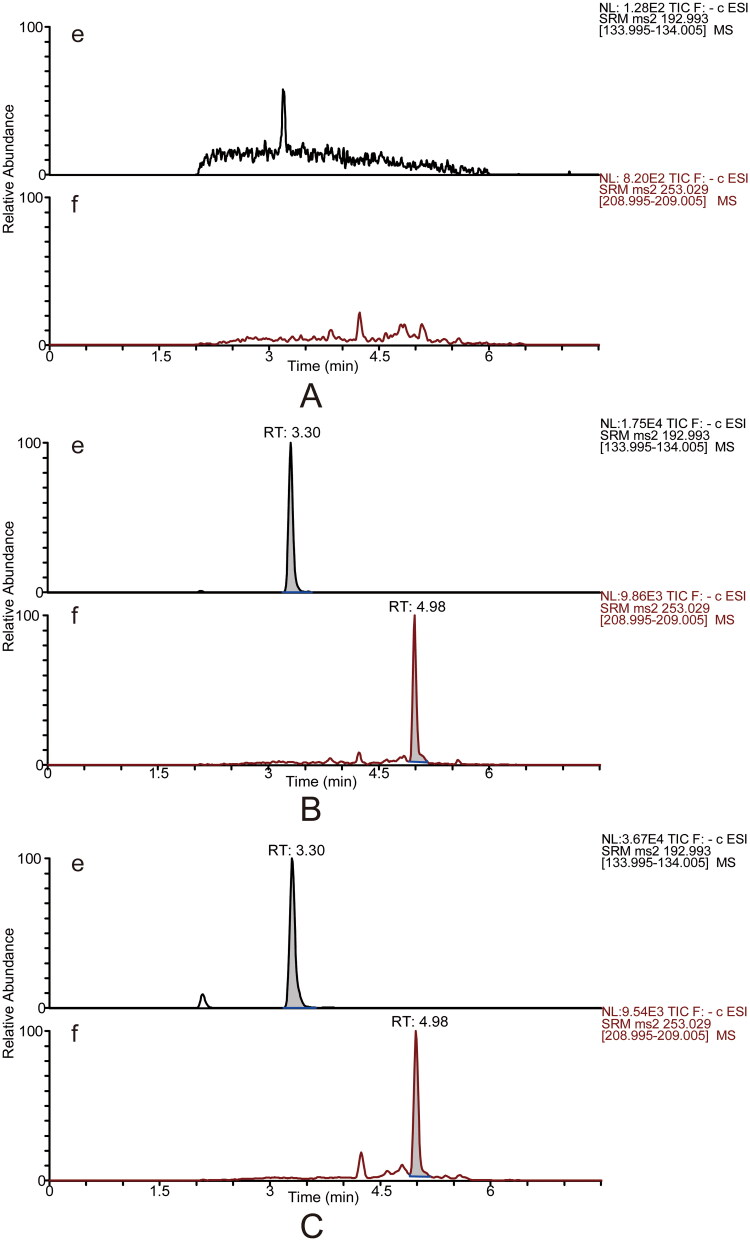
Typical UPLC–MS/MS chromatograms in the negative mode for determining ferulic acid (e) and the internal standard ketoprofen (f, IS2). Blank plasma sample (A). Blank plasma sample spiked with reference standards (medium level QC sample) and the internal standard (B). The rat plasma sample was collected at 5 min after DBD administration (C).

Six individual blank plasma samples were analysed to evaluate the methods’ selectivity. The MRM mode permitted the detection of calycosin-7-*O*-β-d-glucoside, ononin, ligustilide, ferulic acid and the ISs with high selectivity, and there was no interference from endogenous substances. Typical MRM chromatograms of blank plasma, blank plasma spiked with reference and ISs, and plasma samples after single oral administration of DBD are presented in Figures 2 and 3.

The concentration range for calycosin-7-*O*-β-d-glucoside and ononin was 0.5–50 ng/mL; for ligustilide and ferulic acid, it was 5.0–500 ng/mL (Table S1). These compounds showed good linearity. The lower limits of quantification (LLOQs) for calycosin-7-*O*-β-d-glucoside, ononin, ligustilide and ferulic acid were 0.5, 0.5, 5.0 and 5.0 ng/mL, respectively.

#### Precision, accuracy, matrix effect and recovery

The methods’ precision, accuracy, matrix effect and recovery were assessed at three QC levels, as demonstrated in Table S2. The intra- and inter-batch precision and accuracy were expressed as relative standard deviation (RSD) and relative error (RE) percentages, respectively. Both were within accepted variable limits and did not exceed 15%. The calculated matrix effect values and recoveries were within the accepted ranges of 98.1–155.6% and 103.4–119.8%, respectively. They also exhibited RSD under 15%. Notably, the matrix effect of ferulic acid was comparatively higher than that of the other components. However, the results at low, medium and high concentration levels were comparable. This may be due to the diet rats received, which contained bran with high levels of ferulic acid (Ferri et al. 2020; Tang et al. 2021). This caused a high background of ferulic acid in the blank plasma, resulting in diet-induced interference, which could be corrected by fasting treatment lasting the same duration as the pharmacokinetic study. Our findings indicated that the methods were reproducible and precise when determining rat plasma concentrations.

#### Stability

The short-term (25 °C for 8 h), three freeze–thaw cycles, and post-preparative (4 °C for 24 h) stabilities of calycosin-7-*O*-β-d-glucoside, ononin, ligustilide and ferulic acid in rat plasma were investigated by analysing the QC samples at low and high concentration levels. We found no significant differences in responses between the fresh-prepared samples and the stored samples. These results (Table S3) demonstrated that the samples had good stabilities throughout the storing, processing and detecting procedures.

### Comparative pharmacokinetic study

**Figure 4.**
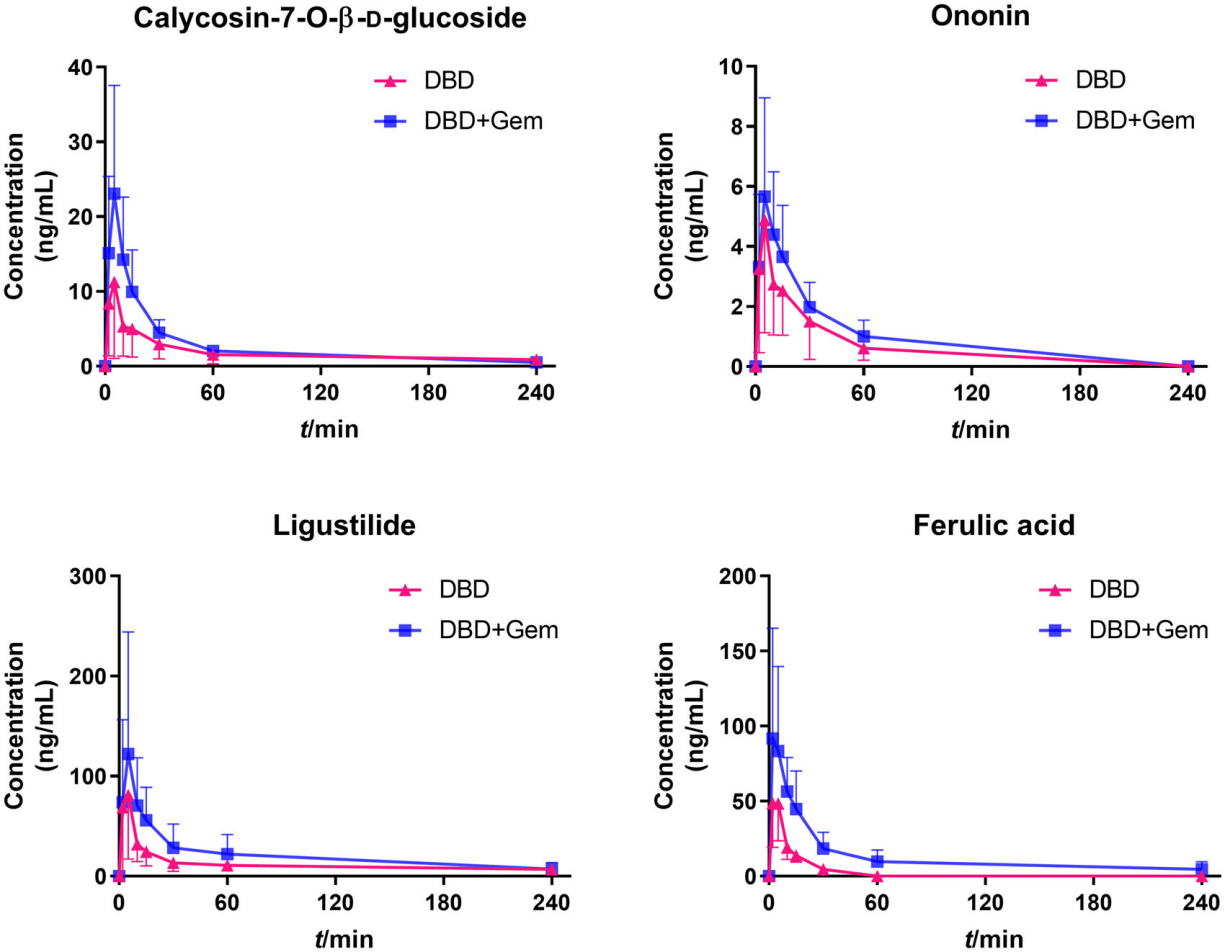
Mean plasma concentration–time profiles of calycosin-7-*O*-β-d-glucoside, ononin, ligustilide and ferulic acid after a single administration of DBD extract and gemcitabine (mean ± SD, *n* = 10).

The validated UPLC–MS/MS methods were utilized to determine the plasma concentrations of calycosin-7-*O*-β-d-glucoside, ononin, ligustilide and ferulic acid at various time points after a single administration of DBD extract or in combination with gemcitabine. The mean plasma concentration–time profiles of the active components are illustrated in Figure 4, and the main pharmacokinetic parameters are shown in Table 2.

**Table 2.** Pharmacokinetic parameters of calycosin-7-*O*-β-d-glucoside, ononin, ligustilide and ferulic acid in rat plasma after a single administration of Danggui Buxue Decoction extract and gemcitabine (mean ± SD, *n* = 10).

Analytes	Calycosin-7-O-β-d-glucoside		Ononin		Ligustilide		Ferulic acid	
	DBD	DBD + Gem	DBD	DBD + Gem	DBD	DBD + Gem	DBD	DBD + Gem
*C*_max_ (ng/mL)	12.1 ± 9.6	23.2 ± 13.8*	5.2 ± 3.5	6.0 ± 3.2	100.8 ± 37.0	133.5 ± 43.4*	54.4 ± 26.8	102.8 ± 43.8*
*T*_max_ (min)	5.1 ± 3.6	5.5 ± 1.5	7.9 ± 8.1	10.5 ± 7.6	5.4 ± 3.4	8.2 ± 4.8	5.5 ± 4.9	6.3 ± 4.4
*t*_1/2_ (min)	70.6 ± 18.7	68.8 ± 15.8	24.0 ± 10.9	26.6 ± 7.1	43.6 ± 9.7	103.2 ± 23.7**	13.0 ± 8.1	101.3 ± 37.6**
AUC_0–_*_t_* (ng/mL × min)	401.3 ± 66.1	624.6 ± 73.1*	107.9 ± 35.9	146.9 ± 45.0*	1664.6 ± 497.0	5090.4 ± 918.6**	614.1 ± 130.5	2879.3 ± 890.0**
AUC_0–∞_ (ng/mL × min)	421.3 ± 61.4	744.9 ± 103.2*	127.2 ± 37.8	185.0 ± 61.2*	2213.8 ± 964.2	6209.2 ± 949.2**	816.7 ± 212.6	4135.5 ± 761.2**
MRT_0–_*_t_* (min)	44.0 ± 27.4	40.0 ± 28.1	16.6 ± 5.6	19.4 ± 5.3	18.9 ± 14.3	44.9 ± 26.6*	7.6 ± 1.8	61.3 ± 30.2**
MRT_0–∞_ (min)	85.8 ± 69.2	76.0 ± 70.4	31.8 ± 16.4	38.3 ± 21.9	34.6 ± 25.8	91.5 ± 55.0*	14.2 ± 10.8	187.9 ± 142.2**
CL/F (mL/min)	591.5 ± 175.0	542.0 ± 128.3	1426.1 ± 595.8	1171.1 ± 403.9	806.1 ± 347.6	204.2 ± 90.1***	212.1 ± 71.3	55.2 ± 31.2***

* *p* < 0.05, DBD + Gem vs. DBD.

** *p* < 0.01, DBD + Gem vs. DBD.

*** *p* < 0.001, DBD + Gem vs. DBD.

All four active components were rapidly absorbed within 10 min, with no significant differences in *T*_max_ between the DBD and DBD + Gem groups. After combination with gemcitabine, among all the pharmacokinetic parameters of ononin, only AUC showed an obvious rise of about 1.36-fold (*p* < 0.05), which was 146.9 ± 45.0 ng/mL × min compared with 107.9 ± 35.9 ng/mL × min in DBD group. The *C*_max_ and AUC_0–_*_t_* of calycosin-7-*O*-β-d-glucoside were 12.1 ± 9.6 ng/mL, 401.3 ± 66.1 ng/mL × min in DBD-administrated rats and 23.2 ± 13.8 ng/mL, 624.6 ± 73.1 ng/mL × min in the combination group, showing an about 1.92-fold and 1.56-fold increase (*p* < 0.05), respectively, indicating the absorption promotion. Pharmacokinetic parameters of ligustilide and ferulic acid were remarkably altered by gemcitabine co-administration, as higher *C*_max_ and AUC with prolonged *t*_1/2_ and MRT, demonstrating absorption and elimination were both affected. The *C*_max_ and AUC_0–_*_t_* of ligustilide were found to be 133.5 ± 43.4 ng/mL, 5090.4 ± 918.6 ng/mL × min in the DBD + Gem group, which were increased by 1.32-fold (*p* < 0.05) and 3.06-fold (*p* < 0.01) as compared to the DBD group. The *t*_1/2_ and MRT of ligustilide were 103.2 ± 23.7 min and 44.9 ± 26.6 min, which were significantly extended by about 2.37-fold (*p* < 0.01) and 2.38-fold (*p* < 0.05) after combination, respectively. The CL/F of ligustilide was 204.2 ± 90.1 mL/min after combination with gemcitabine, showing about 3.95-fold reduction (*p* < 0.001) compared with DBD administration alone. The *C*_max_ and AUC_0–_*_t_* of ferulic acid were 102.8 ± 43.8 ng/mL, 2879.3 ± 890.0 ng/mL × min in the co-administration group, as 54.4 ± 26.8 ng/mL and 614.1 ± 130.5 ng/mL × min compared to the DBD group, indicating about 1.89-fold (*p* < 0.05) and 4.67-fold (*p* < 0.01) enhancement. The *t*_1/2_ and MRT of ferulic acid were found to be 101.3 ± 37.6 min and 61.3 ± 30.2 min, significantly prolonged by about 7.79-fold and 8.06-fold (*p* < 0.01). The clearance of ferulic acid in the DBD + Gem group was 55.2 ± 31.2 mL/min compared with 212.1 ± 71.3 mL/min in the DBD group, indicating a 3.84-fold decline (*p* < 0.001).

### Evaluation of intestinal permeability, transporter and hepatic microsomal enzyme expression

#### Histopathological examination of intestinal permeability and apoptosis analysis of intestinal endothelial cells

**Figure 5.**
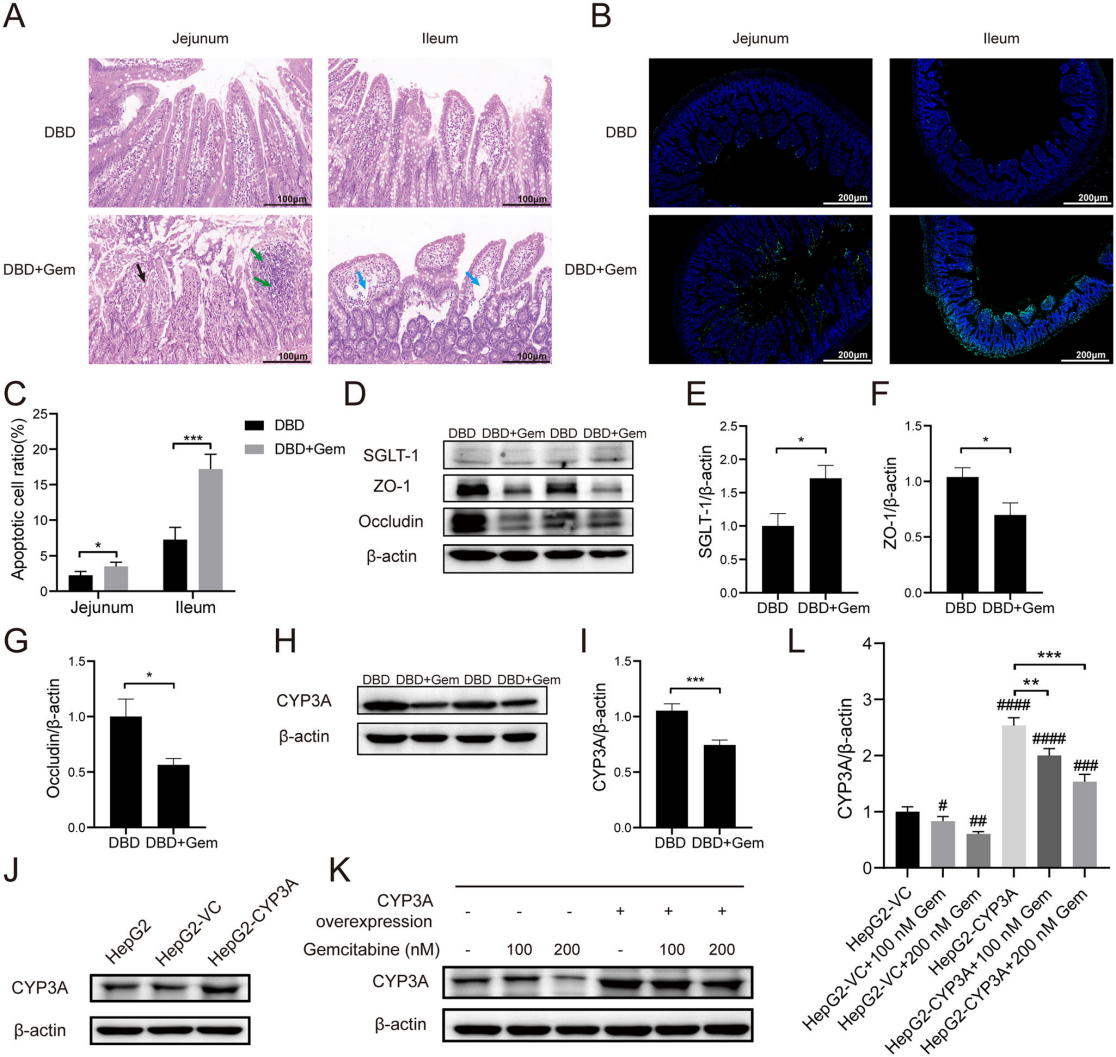
Gemcitabine treatment increased intestinal permeability, up-regulated the expression of SGLT-1, and down-regulated ZO-1, occludin and CYP3A. Haematoxylin–eosin staining histopathological evaluation of jejunum and ileum (A). The microscopic scale bar represents 100 μm. Typical fluorescence images of TUNEL staining in rats’ jejunal and ileal epithelium (B). The scale bar represents 200 μm. The percentage of apoptotic cells in the intestinal endothelial cells of rats (C). *p < 0.05, ***p < 0.001, jejunum vs. ileum. The expressions of SGLT-1, ZO-1 and occludin in the small intestine tissues (D–G). The expression level of CYP3A enzyme in the liver tissues (H, I). *p < 0.05, vs. DBD, ***p < 0.001, vs. DBD. CYP3A was introduced into HepG2 cells, and the overexpression efficacy was confirmed by western blot (J). Western blot typical bands of CYP3A and internal reference β-actin in transfected HepG2 cells (K). The CYP3A/β-actin ratio of HepG2 cells and the overexpression of CYP3A can block the down-regulation of CYP3A expression caused by gemcitabine (L). ^#^*p* < 0.05, vs. HepG2-VC, ^##^*p* < 0.01, vs. HepG2-VC, ^###^*p* < 0.001, vs. HepG2-VC, ^####^*p* < 0.0001, vs. HepG2-VC, **p < 0.01, vs. HepG2-CYP3A, ***p < 0.001, vs. HepG2-CYP3A. The data were expressed as mean ± SEM (*n* = 6).

Haematoxylin–eosin staining was used to examine small intestine histopathology after gemcitabine administration. No apparent abnormalities were observed in the DBD group. In the DBD + Gem group, necrosis, detachment of mucosal epithelial cells, and nuclear pyknosis with hyperchromasia, fragmentation or dissolution of nuclei were observed in the jejunum (Figure 5(A), black arrow). Partial necrosis and dissolution at the top of intestinal villi and focal infiltration of lymphocytes in the lamina propria were occasionally seen in the jejunum (green arrow). In contrast, the ileum showed fewer histological changes, with loosely arranged connective tissue in the lamina propria of the intestinal villi and capillary dilation after gemcitabine administration (Figure 5(A), blue arrow). In addition, gemcitabine significantly increased apoptosis levels (green fluorescence) in rat small intestinal endothelial cells, as seen in Figure 5(B,C). Enhancing apoptosis levels in rats’ intestinal endothelial cells possibly promoted intestinal permeability (Sun et al. 1998; Nakajima et al. 2007). Consequently, gemcitabine could alter the small intestine’s structure with increased endothelial cell apoptosis and intestinal permeability.

#### Protein expressions of SGLT-1, occludin, ZO-1 and CYP3A

The pharmacokinetics of the primary components of DBD was considerably modified, indicating possible alterations in related transporters, drug-metabolizing enzymes and intestinal permeability in rats. Calycosin-7-*O*-β-d-glucoside absorption in the small intestine is mediated by SGLT-1 (Shi et al. 2016), while ligustilide and ferulic acid metabolism are catalysed by CYP3A in the liver (Zhao et al. 2004; Yan et al. 2008; Qian et al. 2009; Zhuang et al. 2017). The permeability of the small intestine could be characterized by the expressions of tight junction proteins ZO-1 and occludin (Zhang and Guo 2009; Ahrne and Hagslatt 2011). Therefore, to examine the impact of combined gemcitabine administration on transporters, metabolic enzymes and intestinal permeability, western blots were utilized to evaluate the expression of SGLT-1, ZO-1 and occludin in the small intestine and CYP3A in the liver.

The protein expression levels of SGLT-1, occludin and ZO-1 in small intestine tissues are depicted in Figure 5(D–G). The administration of gemcitabine significantly increased the SGLT-1 level compared to the DBD group (*p* < 0.05, Figure 5(E)). In contrast, the expression levels of occludin and ZO-1 were notably lower than in the DBD group (*p* < 0.05, Figure 5(F,G)). The relative protein expression of CYP3A in the liver is presented in Figure 5(H,I), demonstrating that gemcitabine prominently down-regulated CYP3A expression compared to the DBD group (*p* < 0.001, Figure 5(I)). This down-regulation effect of gemcitabine on CYP3A expression was further verified in HepG2 cells overexpressing CYP3A, as presented in Figure 5(J–L). Transfection with the PHY-028 vehicle control (VC) vector did not affect CYP3A expression. However, transfection with the PHY-028-CYP3A plasmid significantly up-regulated CYP3A expression level in HepG2 cells (Figure 5(J)) and blocked the down-regulation effect on CYP3A expression caused by gemcitabine in HepG2 cells. Nevertheless, treatment with 200 nM gemcitabine resulted in a significantly lower CYP3A expression level when compared to HepG2 cells overexpressing CYP3A (*p* < 0.001, Figure 5(K,L)).

## Discussion

Gemcitabine is a commonly used medication for the treatment of NSCLC. It has been reported that DBD can enhance the antitumour effect of gemcitabine (Sun et al. 2019; Liu et al. 2021) and can be utilized as adjuvant therapy (Du et al. 2009). Due to the complicated interactions between TCMs and chemotherapeutic drugs, there is a need for systematic research on the interaction between DBD and gemcitabine. A pharmacokinetic study is the crucial foundation for comprehending the herb–drug interaction.

Calycosin-7-*O*-β-d-glucoside, ononin, astragaloside IV, ligustilide and ferulic acid are high-quantity components of DBD that exhibit vital biological activity (Fu et al. 2014; Chiu et al. 2017; Kilani-Jaziri et al. 2017; Lang et al. 2018; Zduńska et al. 2018). Calycosin-7-*O*-β-d-glucoside, astragaloside IV and ferulic acid are also key components listed in Chinese Pharmacopoeia for QC of Radix *Astragalus mongholicus* and Radix *Angelica sinensis* (Chinese Pharmacopoeia Commission 2020). Examining the *in vivo* process of these crucial ingredients of DBD after combination with gemcitabine will facilitate the exploration of the herb–drug interaction.

We conducted a comparative pharmacokinetic study on the active components in DBD before and after combination with gemcitabine, using validated UPLC–MS/MS methods and targeting astragaloside IV, calycosin-7-*O*-β-d-glucoside, ononin, ligustilide and ferulic acid. However, due to the comparatively low plasma concentrations of both the DBD and DBD + Gem groups, only a few concentration–time curves were obtained for astragaloside IV, as a result, pharmacokinetic parameters for this component were no longer calculated. Nevertheless, we analysed and compared the pharmacokinetic behaviours of the other four main components. Following literature reports, alterations in the pharmacokinetics of TCM components may result from the modulation of gastrointestinal function, transporters and metabolic enzymes by chemical drugs in the process of absorption, distribution, metabolism and excretion (Fasinu et al. 2012; Rehman et al. 2015; Zhang et al. 2020; Shang et al. 2021). Consequently, we performed a histological and molecular biological evaluation of intestinal permeability, transporters and hepatic microsomal enzymes to explore the mechanism of the herb–drug interaction between DBD and gemcitabine.

The pharmacokinetic parameters of calycosin-7-*O*-β-d-glucoside, ononin, ligustilide and ferulic acid were significantly altered when combined with gemcitabine. All active constituents of DBD detected in rat plasma were promptly absorbed *in vivo*, reaching *C*_max_ within 10 min. Calycosin-7-*O*-β-d-glucoside and ononin, exhibiting similar chemical structures, displayed comparable pharmacokinetic patterns with significant increases in their AUC in the gemcitabine combination group. The *C*_max_ of calycosin-7-*O*-β-d-glucoside was increased by approximately twofold (*p* < 0.05) when combined with gemcitabine. This compound is absorbed and disposed of in the intestine and transported into small intestinal cells by SGLT-1 (Shi et al. 2016). Previous studies have shown that treatment with gemcitabine can promote glucose uptake (Haberkorn et al. 1994) and enhance glucose transport (Haberkorn et al. 1997). Thus, we analysed the expression of SGLT-1 in the small intestine to investigate the potential mechanism for the observed increase in the bioavailability of calycosin-7-*O*-β-d-glucoside. Our results demonstrated that gemcitabine treatment promoted glucose transporter expression (Figure 5(D,E)), which could explain the reinforced bioavailability of calycosin-7-*O*-β-d-glucoside.

Regarding ligustilide and ferulic acid, the pharmacokinetic parameters were remarkably altered upon the combination with gemcitabine, including higher *C*_max_, *t*_1/2_, AUC, MRT (*p* < 0.05) and declined CL/F (*p* < 0.001), indicating that gemcitabine can accelerate absorption and delay clearance. Ligustilide is primarily absorbed in the intestine, while hepatic elimination is the key route of excretion (Yan et al. 2008). The metabolism of ligustilide is initiated by hepatic cytochrome P450 enzyme CYP3A and other isoenzymes (Yan et al. 2008; Qian et al. 2009). Ferulic acid is predominantly absorbed in the stomach and small intestine (Shi et al. 2014) and metabolized in the liver through CPY3A (Zhao et al. 2004; Zhuang et al. 2017). As gemcitabine can stimulate the intestine, we examined intestinal permeability and observed increased apoptosis levels and reduced protein expressions of ZO-1 and occludin (Figure 5(A–D,F,G)). It has been reported that CYP3A exhibits a good drug-binding affinity for gemcitabine (Subhani and Jamil 2015). In this study, we demonstrated that gemcitabine administration down-regulated CYP3A expression (Figure 5(H,I,K,L)). The down-regulation of ZO-1 and occludin expressions in the intestine and the down-regulation of hepatic CYP3A expression were the primary factors responsible for the altered pharmacokinetic behaviours of ligustilide and ferulic acid.

DBD’s antioxidant (Fu et al. 2014; Zduńska et al. 2018) and immunoregulatory (Yang et al. 2014) properties can enhance the efficacy of gemcitabine while reducing side effects. Among the components with altered pharmacokinetics, ferulic acid was found to increase leukocytes and platelet counts in myelosuppression mice and increase the serum concentrations of GM-CSF and G-CSF, which are frequently used to treat chemotherapy-induced myelosuppression (Ma et al. 2011; Xu 2019). Additionally, due to the herb–drug interaction, the effect of DBD in alleviating toxic side effects produced during gemcitabine treatment may be further reinforced by gemcitabine administration. Similarly, when *Salvia miltiorrhiza* Bunge (Lamiaceae) is combined with warfarin, the pharmacological activity of warfarin is enhanced by alterations to its pharmacokinetics (Chan et al. 1995; Wu and Yeung 2010), and bleeding adverse events have been reported with this herb–drug interaction (Fugh-Berman 2000). Further studies are needed to determine if the pharmacodynamic promotion induced by the herb–drug interaction between DBD and gemcitabine will affect the clinical treatment dosage.

**Figure 6.**
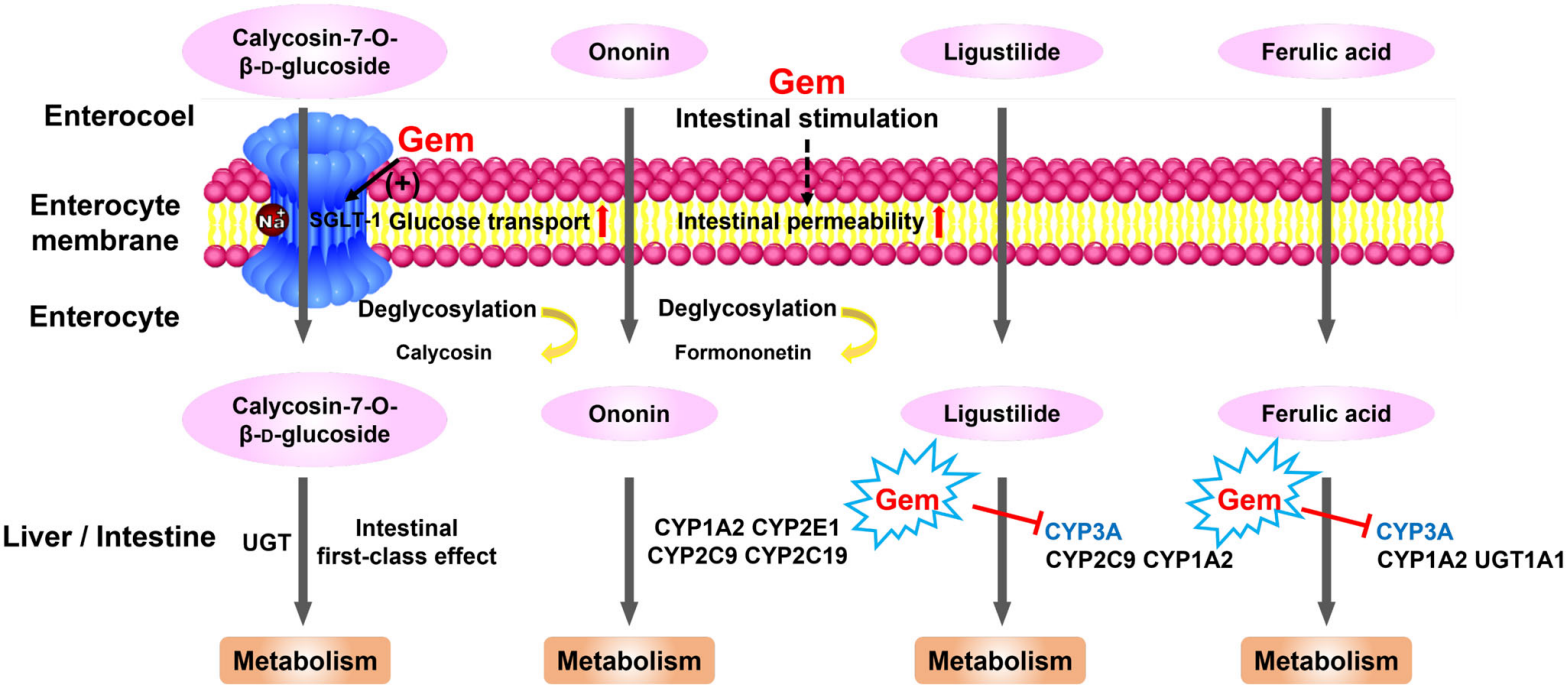
Potential mechanisms through which gemcitabine modified the pharmacokinetic behaviours of active ingredients in DBD by modulating transporters, intestinal permeability and hepatic metabolic enzymes.

In summary, the pharmacokinetic behaviours of active components in DBD were remarkably altered by the co-administration of gemcitabine, leading to increased bioavailability due to promoted intestinal permeability and down-regulated metabolic enzymes (Figure 6). Notably, our study illustrated that gemcitabine interacted with the *in vivo* disposal process of DBD. Additional research conducted by our group demonstrated that DBD could increase the active metabolite of gemcitabine, indicating a reliable herb–drug interaction between DBD and gemcitabine (Sun et al. 2019). Consequently, co-administration of gemcitabine and DBD may enhance the antitumour therapeutic effect of gemcitabine via the increased absorption of phytochemical components.

## Conclusions

This study compared the pharmacokinetics of the four major constituents in DBD, namely, calycosin-7-*O*-β-d-glucoside, ononin, ligustilide and ferulic acid, with and without gemcitabine administration. Our results demonstrated that gemcitabine significantly enhanced the pharmacokinetic exposure of these components. In terms of mechanism, our study revealed that gemcitabine reinforced intestinal permeability by down-regulating the expressions of ZO-1 and occludin and down-regulated hepatic microsomal CYP3A expression. Both effects led to enhanced exposure *in vivo*. These findings provide valuable information on the pharmacokinetic herb–drug interaction between DBD and gemcitabine. Overall, these results could be applied in clinical practice to support the use of DBD as an auxiliary treatment with gemcitabine.

## Supplementary Material

Supplemental Material
