# Supplementary material for: Gemcitabine enhances pharmacokinetic exposure of the major components of Danggui Buxue Decoction in rat via the promotion of intestinal permeability and down-regulation of CYP3A for combination treatment of non-small cell lung cancer
Source: Pharm Biol. 2023 Aug 22;61(1):1298–309. doi: 10.1080/13880209.2023.2246500 (PMC10446811; doi:10.1080/13880209.2023.2246500)
Supplement: Supplemental Material [file IPHB_A_2246500_SM3788.docx]

#### Table S1. Regression data and LLOQs of DBD constituents.

| Analytes | Linear range  (ng/mL) | Linear regression equation | Correlation coefficient  (r) | LLOQs  (ng/mL) |
| --- | --- | --- | --- | --- |
| Calycosin-7-O-β-d-glucoside | 0.50–49.7 | *Y* = 0.0609*C* + 2.26 × 10^-3^ | 0.9992 | 0.50 |
| Ononin | 0.50–49.5 | *Y* = 0.0654*C* + 2.37 × 10^-3^ | 0.9993 | 0.50 |
| Ligustilide | 5.08–508 | *Y* = 0.0413*C* + 2.47 × 10^-3^ | 0.9985 | 5.17 |
| Ferulic acid | 5.03–503 | *Y* = 0.0146*C* + 2.05 × 10^-2^ | 0.9976 | 5.11 |

#### Table S2. Precision, accuracy, matrix effect, and recovery of DBD components (n = 6).

| Analytes | Spiked  (ng/mL) | Precision (%, RSD) | | Accuracy  (%, RE) | Matrix  (%) | Recovery  (%) |
| --- | --- | --- | --- | --- | --- | --- |
|  |  | Intra-batch | Inter-batch |  |  |  |
| Calycosin-7-O-β-d-glucoside | 1.00 | 5.9 | 10.1 | -0.2 | 109.4 ± 6.1 | 118.0 ± 4.9 |
|  | 5.00 | 4.0 | 2.5 | -1.2 | 106.1 ± 15.8 | 114.2 ± 3.2 |
|  | 40.0 | 2.5 | 6.5 | -0.7 | 108.4 ± 3.7 | 119.8 ± 4.1 |
| Ononin | 1.00 | 7.9 | 11.9 | 0.7 | 98.1 ± 2.2 | 108.1 ± 3.4 |
|  | 4.98 | 3.8 | 1.0 | -0.5 | 102.4 ± 13.7 | 103.4 ± 0.8 |
|  | 39.8 | 3.0 | 9.5 | 2.0 | 102.6 ± 2.7 | 110.2 ± 3.8 |
| Ligustilide | 10.3 | 3.8 | 8.6 | -2.9 | 110.8 ± 6.3 | 107.9 ± 2.8 |
|  | 51.7 | 2.2 | 7.1 | -5.7 | 113.4 ± 12.1 | 107.3 ± 2.9 |
|  | 414 | 1.6 | 4.9 | 2.5 | 106.6 ± 4.5 | 106.4 ± 2.1 |
| Ferulic acid | 12.8 | 6.3 | 11.4 | 2.3 | 153.6 ± 9.5 | 106.0 ± 4.1 |
|  | 40.9 | 3.7 | 4.6 | 6.9 | 155.6 ± 9.0 | 108.8 ± 1.9 |
|  | 307 | 3.8 | 9.2 | -1.4 | 139.8 ± 6.6 | 107.0 ± 1.5 |

#### Table S3. Stabilities of DBD components in rat plasma under different conditions (RE%, n = 3).

| Analytes | Spiked  (ng/mL) | 3 freeze-thaw cycles | Short-term  25°C for 8 h | Post-preparative  4°C for 24 h |
| --- | --- | --- | --- | --- |
| Calycosin-7-O-β-d-glucoside | 1.00 | -1.0 | 13.0 | -9.2 |
|  | 40.0 | -2.5 | -11.0 | 0.9 |
| Ononin | 1.00 | -14.9 | 5.1 | -11.1 |
|  | 39.8 | -2.2 | -11.4 | -0.9 |
| Ligustilide | 10.3 | -13.1 | -4.1 | -6.8 |
|  | 414 | -1.0 | -8.4 | 1.0 |
| Ferulic acid | 12.8 | -8.8 | -12.0 | -11.0 |
|  | 307 | 2.8 | 8.6 | 1.5 |
